# Rapid and Scalable Plant-Based Production of a Potent Plasmin Inhibitor Peptide

**DOI:** 10.3389/fpls.2019.00602

**Published:** 2019-05-15

**Authors:** Mark A. Jackson, Kuok Yap, Aaron G. Poth, Edward K. Gilding, Joakim E. Swedberg, Simon Poon, Haiou Qu, Thomas Durek, Karen Harris, Marilyn A. Anderson, David J. Craik

**Affiliations:** ^1^ Institute for Molecular Bioscience, The University of Queensland, Brisbane, QLD, Australia; ^2^ Department of Biochemistry and Genetics, La Trobe Institute for Molecular Science, La Trobe University, Melbourne, VIC, Australia

**Keywords:** peptide, therapeutic, asparaginyl endopeptidase, cyclyzation, stability, biofactory, *Nicotiana benthamiana*, sunflower trypsin inhibitor

## Abstract

The backbone cyclic and disulfide bridged sunflower trypsin inhibitor-1 (SFTI-1) peptide is a proven effective scaffold for a range of peptide therapeutics. For production at laboratory scale, solid phase peptide synthesis techniques are widely used, but these synthetic approaches are costly and environmentally taxing at large scale. Here, we developed a plant-based approach for the recombinant production of SFTI-1-based peptide drugs. We show that transient expression in *Nicotiana benthamiana* allows for rapid peptide production, provided that asparaginyl endopeptidase enzymes with peptide-ligase functionality are co-expressed with the substrate peptide gene. Without co-expression, no target cyclic peptides are detected, reflecting rapid *in planta* degradation of non-cyclized substrate. We test this recombinant production system by expressing a SFTI-1-based therapeutic candidate that displays potent and selective inhibition of human plasmin. By using an innovative multi-unit peptide expression cassette, we show that *in planta* yields reach ~60 μg/g dry weight at 6 days post leaf infiltration. Using nuclear magnetic resonance structural analysis and functional *in vitro* assays, we demonstrate the equivalence of plant and synthetically derived plasmin inhibitor peptide. The methods and insights gained in this study provide opportunities for the large scale, cost effective production of SFTI-1-based therapeutics.

## Introduction

Beyond its biological role as a plant defense peptide, the 14 amino acid sunflower trypsin inhibitor-1 (SFTI-1) has attracted significant interest in the drug development field (Craik et al., 2006; Lesner et al., 2011). This interest largely stems from the cyclic backbone of SFTI-1, which together with a bridging disulfide bond imparts exceptional stability and conformational rigidity to the peptide (Korsinczky et al., 2001). Furthermore, SFTI-1 is readily tolerant of residue substitutions, exemplified by a wide range of combinatorial and rational design variants that have been applied to convert SFTI-1 into potent and stable inhibitors of therapeutically relevant proteases, including matriptases (Quimbar et al., 2013; Fittler et al., 2014; Gitlin et al., 2015), kallikreins (Shariff et al., 2014; Chen et al., 2016; de Veer et al., 2016; Jendrny and Beck-Sickinger, 2016), chymotrypsin (Swedberg et al., 2017), and furin (Fittler et al., 2015). SFTI-1 has also proven to be a useful scaffold for presenting and stabilizing small bioactive epitopes that by themselves would be unstable and not effective as pharmaceuticals (Chan et al., 2011; Qiu et al., 2017; Durek et al., 2018). These engineered cyclic SFTI-1 analogues uniformly display significantly enhanced serum stability compared with their linear counterparts, overcoming a major limitation of peptide-based therapeutics (Wang and Craik, 2018).

Peptide drugs are primarily produced *via* solid phase peptide synthesis techniques, which in large scale have considerable economic and environmental costs (Andersson et al., 2000). For some peptides, recombinant production is a feasible alternative, with prokaryotes and lower eukaryotic hosts most commonly used (Demain and Vaishnav, 2009). In the case of SFTI-1, backbone cyclization is required for maximum potency (Colgrave et al., 2010), so recombinant production strategies that incorporate this post-translational modification are required. Recently, an intein-mediated protein splicing approach to SFTI-1 cyclization in *E. coli* was reported, with SFTI-1 yields estimated at 180 μg/L of bacterial culture (Li et al., 2016). Although promising, intein splicing efficiency is highly sensitive to the residues at the extein-intein junction, potentially reducing its broad applicability (Aboye and Camarero, 2012). As an alternative, and considering that SFTI-1 is naturally produced and cyclized in sunflower, a plant-based production system is appealing. However, small peptides have typically proven difficult to produce *in planta*, presumably due to the unintended effects of proteolysis either *in planta* or during extraction phases (Benchabane et al., 2008; Habibi et al., 2017). Strategies to overcome this limitation have included expressing peptides with stabilizing fusion partners (Yasuda et al., 2005; Sainsbury et al., 2013), down-regulating interfering plant proteases (Robert et al., 2015), and the development of subcellular targeting approaches (Jackson et al., 2010; Yang et al., 2017). Despite advances using these strategies, the yields from plant-produced peptides have generally been low, typically in the low μg g^−1^ fresh weight (FW) range (Lico et al., 2012; Viana et al., 2012). In contrast, some endogenous cyclic plant peptides are known to accumulate to very high levels [~1.8 mg g^−1^ dry weight (DW)], most notably exemplified by the class of cyclic peptides termed cyclotides (Craik et al., 1999; Seydel and Dornenburg, 2006). Thus, determining the *in planta* biosynthetic pathways that govern cyclotide synthesis and accumulation in plants will be of great benefit if translatable to the recombinant production of “designer” therapeutic peptides.

**Figure 1:**
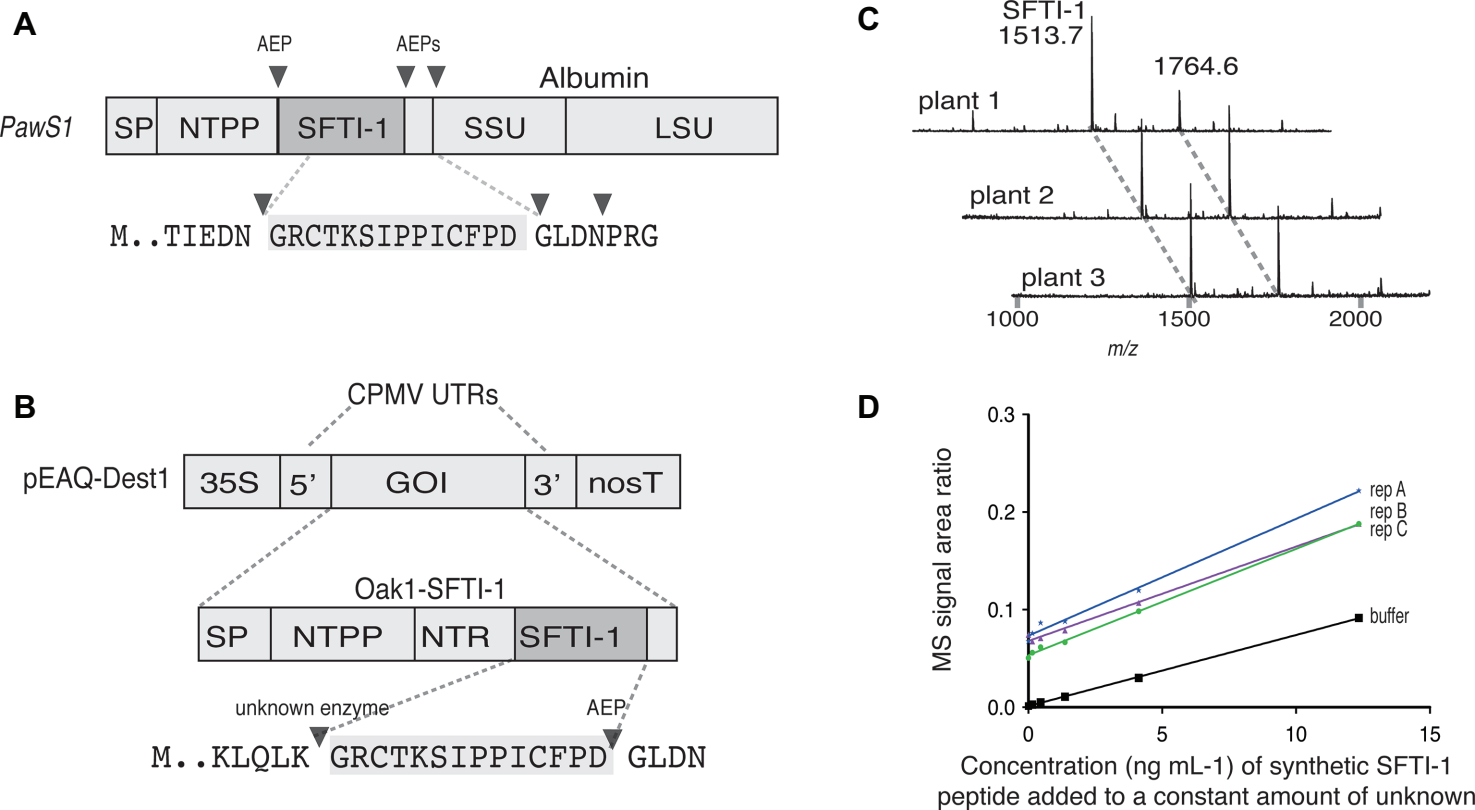
Transient expression analysis of SFTI-1 production in *N. benthamiana*. **(A)** Native SFTI-1 is produced in sunflower seed *via* expression of the *PawS1* gene. SFTI-1 processing occurs *via* the concerted action of asparaginyl endopeptidases (AEPs), which have strict preference for asparagine or aspartic residues (shown as filled triangles). The 14-amino acid SFTI-1 peptide sequence and immediately flanking residues are displayed with the SFTI-1 sequence highlighted in grey. **(B)** For transient expression in *N. benthamiana* leaves, the pEAQ-Dest1 vector (Sainsbury et al., 2009) was used which provides high level transgene expression due the presence of the 35 s promoter and cowpea mosaic virus (CPMV) 5′ and 3′ UTRs. To produce SFTI-1, the *Oak1* gene was engineered to include the SFTI-1 peptide encoding sequence, replacing that of kB1. Cleavage after an amino-terminal repeat (NTR) by an as yet unidentified protease is thought to occur first to liberate the N-terminal glycine required for AEP mediated backbone cyclization to the C-terminal aspartic residue. **(C)** MALDI-TOF MS analysis of peptides produced in *N. benthamiana* leaves upon co-expression of pEAQ-OaAEP1_b_ with pEAQ-Oak1-SFTI-1. The mass for cyclic SFTI-1 (*m/z* 1513.7) was readily detected. An unrelated and endogenous peptide (*m/z* 1764.7) was also readily detected. **(D)** SFTI-1 peptides were quantified using the method of standard addition where a standard curve was built into each crude plant extract.

SFTI-1 is produced in sunflower seeds, where it is post-translationally processed from the precursor protein PawS1 (preproalbumin with sunflower trypsin inhibitor-1) (Mylne et al., 2011) (Figure 1A). Because sunflower transformation is inefficient, most PawS1 processing studies have been done in the model plant Arabidopsis (Mylne et al., 2011), *in situ* using sunflower seed extracts (Bernath-Levin et al., 2015), or *in vitro* with recombinant processing enzymes and synthetic or recombinant substrates (Bernath-Levin et al., 2015; Franke et al., 2017; Haywood et al., 2018). Together these studies have unequivocally demonstrated the involvement of vacuolar cysteine proteases termed asparaginyl endopeptidases (AEPs) for both the cleavage and subsequent cyclization of SFTI-1. Similarly, cyclotides are known to be backbone cyclized by AEPs (Bernath-Levin et al., 2015; Harris et al., 2015; Poon et al., 2017), where a detailed understanding of mechanisms and structural requirements has emerged (Jackson et al., 2018; James et al., 2018). These ligase competent AEPs not only represent useful biotechnological tools for *in vitro* peptide and protein engineering applications (Harris et al., 2015; Nguyen et al., 2015; Hemu et al., 2016) but also open up opportunities for their deployment in plant biofactory applications for the production of cyclic peptides (Poon et al., 2017).

In this study, we evaluated a series of gene expression parameters for optimizing *in planta* SFTI-1 production using *N. benthamiana* as a biofactory host. We demonstrate that resultant yields are influenced by the choice of AEP ligase, the AEP recognition site used, and by using multi-unit peptide expression cassettes. We demonstrate the scalability and usefulness of this transient plant-based production system by producing and purifying a recently developed potent plasmin inhibitor therapeutic based on SFTI-1 (Swedberg et al., 2019).

## Materials and Methods

### Vector Construction

DNA encoding Oak1-SFTI-1_GLDN, Oak1-[D14N]SFTI-1_GLDN, Oak1_GLDN, Oak1-[N29D]_GLDN, Oak1-[T4Y,I7R]SFTI-1, and Oak1-[T4Y,I7R]SFTI-1_3R were synthesized by Integrated DNA Technologies (Singapore) as gene block fragments in preparation for in house cloning (Supplementary Figure S7). All peptide precursor genes and AEP genes were recombined into the plant expression vector pEAQ-DEST1 (Sainsbury et al., 2009) using Gateway™ LR Clonase™ technology (Invitrogen, Carlsbad CA). Sequence verified vectors were then transferred to *Agrobacterium tumefaciens* LBA4404 by electroporation.

### Transient Expression in *Nicotiana benthamiana*

*Nicotiana benthamiana* plants were cultivated in Jiffy peat pellets in a plant growth room at 28°C under 160 μmol of LED illumination (AP67 spectra, Valoya Oy, Helsinki, Finland). Agrobacterium cultures harboring pEAQ-DEST1 expression cassettes were grown in Luria-Bertani media to stationary phase before centrifugation and resuspension in infiltration buffer (10 mM MES)(2-[*N*-morpholino]ethanesulfonic acid) (pH 5.6, 10 mM MgCl_2_, 100 uM acetosyringone). Separate cultures (OD600 of 1.0) harboring the AEP and peptide expression vectors were mixed at a ratio of 1:1 before vacuum infiltration of *N. benthamiana* plants at 5–6 weeks of age. For relative quantification experiments, a third Agrobacterium culture was added to the mix that contained an expression vector encoding a truncated kB6 peptide. This design which allowed only linear kB6 to be produced, irrespective of AEP expression, served as an internal control for normalizing SFTI-1 and kB1 levels.

### Peptide Quantification

At 6 days post-infiltration, plant tissue was harvested for peptide extraction and analysis. To enable absolute quantification of SFTI-1 and [T4Y,I7R]SFTI-1, leaf tissue was lyophilized and ground using a Geno/Grinder® (SPEX Sample Prep) with homogenous subsamples used for MS-based quantification. Routinely, 5 mg of dry tissue was reconstituted in buffer [50% (v/v) acetonitrile, 0.1% (v/v) formic acid] with 0.05 μM codeine included as internal standard. Triplicate samples were spiked with a standard curve of concentrations of analyte before centrifugation. The analytes were quantified using targeted multiple reaction monitoring (MRM) analyses conducted on a SCIEX QTRAP 6500^+^ mass spectrometer interfaced with a SCIEX UPLC system. Studies were conducted using a Phenomenex Kinetex C18 UPLC column (150 mm × 2.0 mm, 1.7 μm particle size), maintained at 60°C with a linear acetonitrile gradient delivered at a flow rate of 0.4 ml min^−1^. Ion spray voltage was set at 5000 V, source temperature at 400°C, and MRM scans were conducted with unit resolution settings for both Q1 and Q3. MRM transition details for each analyte are provided in Supplementary Table S1. SCIEX MultiQuant (v 3.0.2) software was used to plot analyte signal intensities against concentrations added to the sample matrix and back-extrapolated to find negative X-axis intercepts, which were finally adjusted for dilution to calculate analyte concentrations in the sample.

For relative quantification, leaf disks were punched from infiltrated leaves and placed in microfuge tubes with ball bearings, before grinding to powder in liquid nitrogen using a Geno/Grinder® (SPEX Sample Prep). Peptides were extracted in 200 μl of aqueous [50% (v/v) acetonitrile, 1% (v/v) formic acid] with gentle mixing overnight. After centrifugation, the supernatant was diluted 1:5 with 1% formic acid before being desalted and concentrated using C18 ZipTips (Millipore). Samples were then mixed 1:1 with a-cyano-4-hydroxycinnamic acid [5 mg ml^−1^ in 50% acetonitrile, 0.1% TFA, 5 mM (NH_4_)H_2_PO_4_] before being spotted and dried onto a MALDI sample plate for matrix assisted laser desorption/ionization (MALDI)-time of flight (TOF) MS using an Applied Biosystems 4700 TOF-TOF Proteomics Analyzer. For relative quantification, the sum of the isotope cluster area corresponding to cyclic SFTI-1 or kB1 was normalized to the sum of the isotope cluster area of linear kB6 peptides.

### Peptide Synthesis and Purification

All peptides were synthesized in house using established Fmoc solid-phase peptide synthesis methods (Cheneval et al., 2014). Peptides were isolated by RP-HPLC and characterized by high resolution MS and NMR spectroscopy.

### *In vitro* Peptide Cyclization Assays

Recombinant OaAEP1_b_ was prepared using the methods detailed in (Harris et al., 2015) with activated enzyme concentration estimated by BCA assay according to the manufacturer’s instructions. Linear target peptides (6.67 μM) were incubated with recombinant OaAEP1_b_ (7.5 μg ml^−1^ final concentration) in activity buffer (50 mM sodium acetate, 50 mM NaCl, 1 mM EDTA, pH 5) for up to 40 h at room temperature. The reaction mixture (10 μl) was desalted using C18 ZipTips (Millipore) and mixed 1:1 with a-cyano-4-hydroxycinnamic acid [5 mg ml^−1^ in 50% acetonitrile, 0.1% TFA, 5 mM (NH_4_)H_2_PO_4_] before MALDI-TOF MS using an Applied Biosystems 4700 TOF-TOF Proteomics Analyzer.

### Peptide Extraction and Purification

Harvested plant tissue at 6 days post-infiltration was lyophilized then homogenized using a Geno/Grinder® (SPEX Sample Prep) prior to solvent extraction with 50% (v/v) acetonitrile, 0.1% (v/v) formic acid. The supernatant collected was lyophilized, then redissolved with 10% (v/v) acetonitrile, 0.1% (v/v) formic acid before Solid-Phase Extraction (SPE) using a Phenomenex Strata C18-E SPE cartridge with 10 g resin capacity. The eluted fraction of 5–20% (v/v) acetonitrile, 0.1% (v/v) formic acid was then collected, lyophilized, and then reconstituted in 5% (v/v) acetonitrile, 0.1% (v/v) trifluoroacetic acid. The elution was then passed through a 0.45 μm filter before separation to homogeneity by HPLC on a semipreparative Phenomenex Jupiter C18 RP-HPLC column (250 mM × 10 mM, 5 μm particle size) followed by a preparative analytical Phenomenex Jupiter C18 RP-HPLC column (250 mm × 4.6 mm, 5 μm particle size). Fractions yielding homogeneous plant derived [T4Y,I7R]SFTI-1 were identified using MALDI-TOF as described above and lyophilized.

### Structural Characterization of Plant Derived [T4Y,I7R]SFTI-1

High resolution MS comparison of synthetic and plant-produced peptides was conducted *via* UPLC-MS analysis on a SCIEX X500R mass spectrometer interfaced with a SCIEX UPLC. UPLC column details were identical to those used in the QTRAP analyses detailed earlier. A linear acetonitrile gradient was delivered over 15 min (flow rate 0.4 ml min^−1^) and monitored *via* positive ion TOF-MS acquisition (250 ms per scan, *m/z* 100–1,000). Retention times were determined from post-run XICs with a mass extraction width of 0.05 Da centered on monoisotopic peaks.

Prior to tandem MS, plant-derived [T4Y,I7R]SFTI-1 was redissolved in 100 mM NH_4_HCO_3_ (pH 8) for reduction, alkylation, and enzymatic digestion with bovine trypsin (Sigma T1426). Tandem MS of linear reduced and carboxyamidomethylated [T4Y,I7R]SFTI-1 fragment with sequence SRPPICFPDGR was collected on a SCIEX 5600 TripleTOF instrument interfaced with a Shimadzu UPLC. Sample was separated on a Agilent Zorbax 300SB-C18 column (100 mm × 2.1 mm, 1.8 μm particle size) and eluted with a linear acetonitrile gradient, and eluent was monitored using an information dependent acquisition experiment with a TOF-MS survey scan (mass range *m/z* 80–1,000 Da) triggering up to 20 MS/MS on precursor ions (mass range *m/z* 80–1,100 Da) with 50 ms scan times.

### NMR Spectroscopy

The heterologously plant-produced and purified peptide [T4Y,I7R]SFTI-1 was dissolved in 90% H_2_O/10% D_2_O at a concentration of 80 μg ml^−1^. Spectra were recorded on a Bruker Avance III 600 MHz spectrometer equipped with a cryoprobe at 298 K. Phase-sensitive mode using time-proportional phase incrementation for quadrature detection in the t1 dimension was used for all two-dimensional spectra. Excitation sculpting with gradients was used to achieve water suppression. NMR experiments included TOCSY using a MLEV-17 spin lock sequence with an 80-ms mixing time, and NOESY with a 200-ms mixing time. Spectra were recorded with 4,096 data points in the F2 dimension and 512 increments in the F1 dimension. The t1 dimension was zero-filled to 1,024 real data points, and the F1 and F2 dimensions were multiplied by a sine-squared function before Fourier transformation. Chemical shifts were referenced to DSS. All spectra were processed using TopSpin (Bruker) and manually assigned with CCPNMR using the sequential assignment protocol (Wüthrich, 1986; Vranken et al., 2005).

### Plasmin Inhibitory Assays

A serial dilution of plant-derived [T4Y,I7R]SFTI-1 was incubated with 1 nM native human plasmin (Sigma-Aldrich) for 30 min in assay buffer (0.1 M Tris-HCl, pH 8.0, 0.1 M NaCl, and 0.005% Triton X-100) in low binding 96-well plates (Corning). After addition of 100 μM of the colorimetric peptide substrate Acetyl-Arg-Met(sulphone)-Tyr-Arg-*p*NA (*K*_M_ = 23.5 μM) to a final volume of 200 μl, the rate of substrate cleavage was monitored by the release of the *p*NA moiety at *λ* = 405 nm over 7 min. The inhibition constant (*K*_i_) was determined from three independent assays by the Morrison equation and non-linear regression using GraphPad Prism 6.

### Statistical Analysis

One-way ANOVA followed by Tukey’s multiple comparisons test was performed using GraphPad Prism version 7.0c for Mac OS X, GraphPad Software, La Jolla California USA, www.graphpad.com.

### Accession Numbers

AEP gene sequences that have assigned GenBank accession numbers include OaAEP1_b_ (KR259377), CtAEP1 (KF918345), PxAEP3b (MG720076), and HeAEP3 (MG720074).

## Results

### *Nicotiana benthamiana* Leaf-Based Transient Expression of Sunflower Trypsin Inhibitor-1

To express and cyclize SFTI-1 *in planta*, we used the pEAQ vector (Figure 1B) (Sainsbury et al., 2009) for recombinant production in *N. benthamiana*. Initially, to produce native SFTI-1, we tested peptide accumulation in leaves upon expression of a modified *Oak1* gene [described in (Poon et al., 2017)] where the peptide sequence for SFTI-1 replaces the cyclotide peptide kalata B1 (kB1) (construct pEAQ-Oak-SFTI-1) (Figures 1A,B). In addition, we chose to replace the C-terminal GLPSLAA residues normally present in Oak1 with the residues GLDN that naturally flank SFTI-1 within the PawS1 precursor protein. As previously shown (Poon et al., 2017), cyclic SFTI-1 (*m/z* 1513.7) could only be detected in leaf extracts upon co-expression of the SFTI-1 precursor with the ligase-efficient AEP from *O. affinis* (OaAEP1_b_) (Figure 1C). To quantify the yield of SFTI-1 produced in *N. benthamiana* leaves, we used a quantitative mass spectroscopy (MS)-based approach (Bronsema et al., 2012) (Figure 1D). This approach, which requires a standard curve to be included in each replicate extraction, provides for a more accurate measurement by eliminating any effect that sample matrix might have on SFTI-1 signal intensity. Using this method, the yield of cyclic SFTI-1 extracted was determined to be 12.8 ± 3.0 μg g^−1^ DW (s.d., *n* = 3), which is substantially lower than the reported 199 μg g^−1^ DW yield obtained for the cyclotide kB1, using a similar expression strategy (Poon et al., 2017).

**Figure 2:**
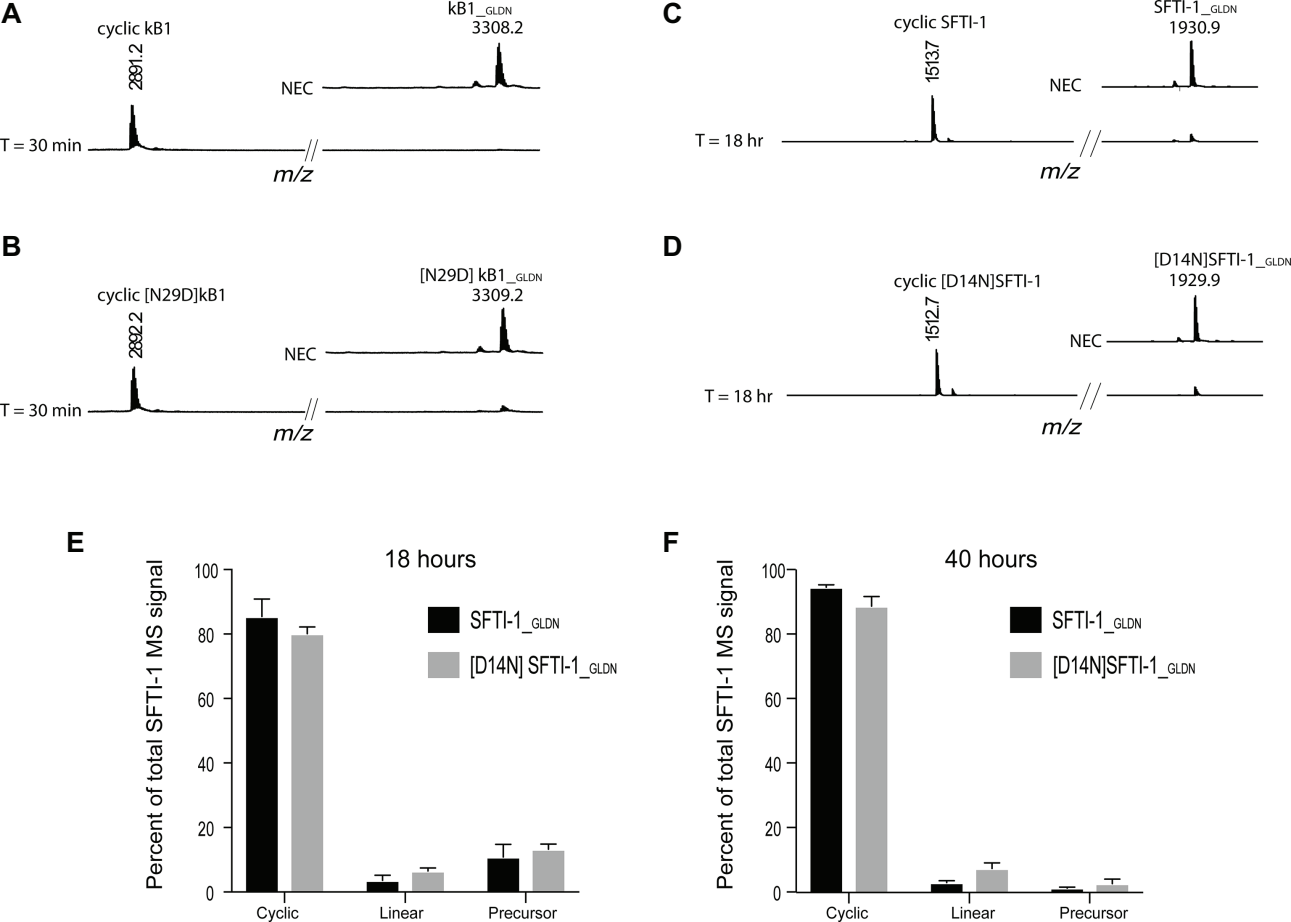
*In vitro* AEP cyclization efficiency. Representative MALDI-TOF-MS traces of the processing products of precursors **(A)** kB1__GLDN_, **(B)** [N29D]kB1__GLDN_, **(C)** SFTI-1__GLDN_, and **(D)** [D14N]SFTI-1__GLDN_. Both kB1 substrates were essentially quantitatively converted to cyclic forms within 30 min, while SFTI-1 substrates were still detectable after 18 h of reaction time. NEC, no enzyme control. Percent of total SFTI-1 and [D14N]SFTI-1 MS signal for cyclic, linear, and precursor form after 18 **(E)** and 40 h **(F)** of incubation (*n* = 6). Peptide precursor (6.67 μM final) was incubated at pH 5.0 with recombinant OaAEP1_b_ enzyme at a final concentration 7.5 μg ml^−1^.

To determine if this lower *in planta* SFTI-1 yield correlates with a lower efficiency of OaAEP1_b_ on SFTI-1 substrates, we set out to compare processing efficiencies between SFTI-1 and kB1 substrates *in vitro* (Figure 2). AEPs are known to have strict preference for either an Asn or Asp at the P1 position, thus we additionally wished to determine the effect that reciprocal Asn/Asp residue changes would have on processing efficiencies. For this, we directly compared recombinant OaAEP1_b_ activity on the peptide substrates kB1__GLDN_ (Figure 2A), [N29D] kB1__GLDN_ (Figure 2B), SFTI-1__GLDN_ (Figure 2C), and [D14N]SFTI-1__GLDN_ (Figure 2D). Peptide cyclization assays were performed at pH 5.0 to simulate the low pH of leaf cell vacuoles, where AEP-mediated cyclization is predicted to occur (Jackson et al., 2007; Conlan et al., 2011). For all substrates, peptide cyclization was favored over hydrolysis by recombinant OaAEP1_b_ with resulting MS signals for cyclic peptide dominating over linear peptide. For both kB1 substrates, the precursor was essentially quantitatively converted to cyclic kB1/[N29D] kB1 within 30 min (Figures 2A,B), while for the SFTI-1__GLDN_ and [D14N]SFTI-1__GLDN_ precursor peptides, unprocessed peptides were still detectable after 18 h (Figures 2C–E). Further analysis after 40 h of incubation revealed that processing was essentially complete with resulting cyclic to linear peptide MS signal ratios calculated at 96.86 ± 0.66% (s.d., *n* = 6) and 92.19 ± 1.89% (s.d., *n* = 6) for SFTI-1__GLDN_ and [D14N]SFTI-1__GLDN_, respectively (Figure 2F). These findings indicate that SFTI-1 precursor peptides, irrespective of containing Asp or Asn at the AEP processing site, are although amenable to enzymatic cyclization, relatively poor substrates for OaAEP1_b_ with significantly slower processing when compared to kB1 cyclotide precursors. Thus, we reasoned that the lower *in planta* yields observed for SFTI-1 over kB1 may be caused by OaAEP1_b_ being outcompeted for SFTI-1 substrates by endogenous AEPs, which lack the ability to stabilize SFTI-1 by way of backbone cyclisation. Optimization or discovery of AEP ligases more conducive to SFTI-1 substrates, or downregulation of interfering endogenous AEP machinery are thus two approaches to increase the *in planta* yield of SFTI-1.

### Assaying Diverse Asparaginyl Endopeptidases for *in planta* Cyclization of Sunflower Trypsin Inhibitor-1

**Figure 3:**
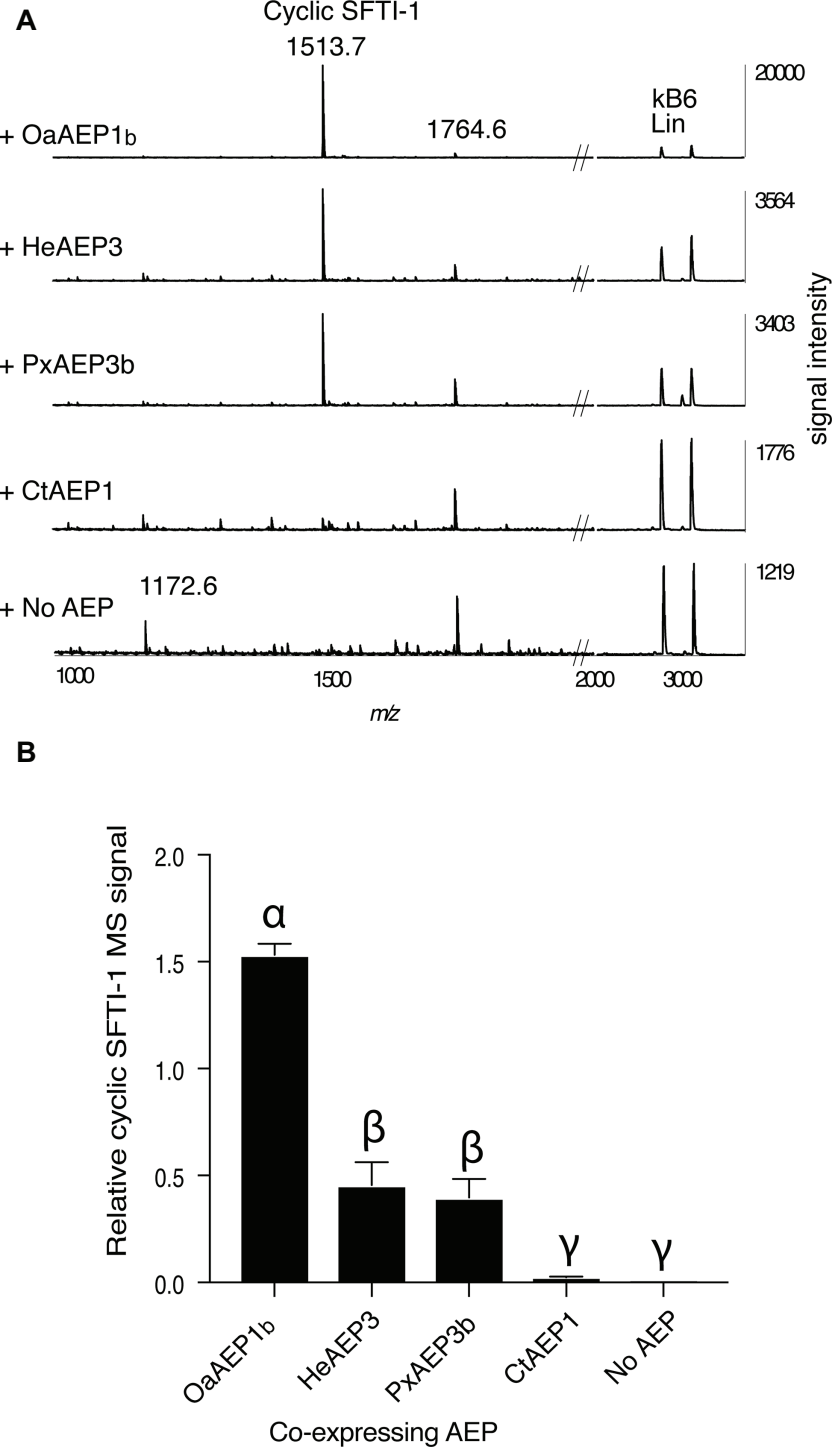
Comparison of AEP ligases for the *in planta* SFTI-1 peptide cyclization. **(A)** MALDI-TOF MS analysis of representative (*n* = 3) peptide extracts from *N. benthamiana* leaves which were co-infiltrated with pEAQ-Oak1-SFTI1_GLDN and pEAQ-Oak6_trun with or without pEAQ driven AEP ligase genes. Without AEP transgene expression, no cyclic or full length linear SFTI-1 related peptides were detectable. Smaller masses, however, were observed, with low signal strengths, and likely represent truncated SFTI-1 peptides (e.g., *m/z* 1172.6 consistent with linear oxidized GRCTKSIPPIC. By co-expressing AEP ligase genes from *O. affinis* (OaAEP1_b_), *H. enneaspermus* (HeAEP3) and Petunia “Mitchell” (PxAEP3b) cyclic SFTI-1 was readily detected which contrasted to expression of CtAEP1 which failed to produce any cyclic SFTI-1. For all infiltrations, the expression of pEAQ-Oak6_trun served as an internal control where MS signal intensities for linear kB6 were used to normalize SFTI-1 MS signals for relative quantification. **(B)** Relative MS signal intensities for cyclic SFTI-1 among co-expressed ligase capable AEPs (*n* = 3). Treatments carrying unique Greek lettering are significantly different (*p* < 0.05) as determined by Tukey’s ANOVA. Error bars are s.e.m.

In addition to OaAEP1_b_ from *O. affinis* (Harris et al., 2015), several other AEP ligases have recently been characterized, including HeAEP3 (*Hybanthus enneaspermus*) (Jackson et al., 2018), PxAEP3b (petunia) (Jackson et al., 2018), and CtAEP1 (butelase-1; *Clitoria ternatea*) (Nguyen et al., 2015). To determine if any of these newly discovered AEP ligases have improved *in planta* processing ability for SFTI-1 over OaAEP1_b_, we set up an *in planta* assay wherein the precursor gene Oak1-SFTI-1, the AEP gene in question, and a C-terminally truncated kB6 peptide precursor gene were co-expressed (Figures 3A,B). Expression of the latter construct resulted in the production of a kB6 peptide precursor without the required C-terminal residues for AEP-mediated cyclization. Thus, the accumulation level of linear kB6 could serve as an internal control to normalize for differences in infiltration efficiencies between *N. benthamiana* leaves. Importantly, this approach produced very similar results to that obtained using normalization with a spiked peptide on a per dry weight basis (Supplementary Figure S1). Of the AEPs tested, OaAEP1_b_ from *O. affinis* proved to be the best performing peptide ligase for SFTI-1 with a ~3 fold increase in relative abundance, when compared to the next best performing ligases, HeAEP3 and PxAEP3b, which yielded similar SFTI-1 levels (Figures 3A,B). CtAEP1 (butelase-1) produced only minimal cyclic SFTI-1 in agreement with the *in vitro* characterization of this enzyme as inefficient for cyclisation at Asp residues (Nguyen et al., 2015).

Co-expression of AEPs with *Oak1* (that harbors an asparagine at the cyclization site of kB1) revealed that CtAEP1 produced the highest relative level of kB1 when compared to OaAEP1_b_, HeAEP3, and PxAEP3b (Supplementary Figures S2A,C). With this in mind, we wished to determine if cyclization levels of SFTI-1 could be simply improved by combining CtAEP1 expression with a modified SFTI-1 where the cyclization residue was changed from the native aspartic acid to an asparagine (construct pEAQ-Oak1-[D14N]SFTI-1). Somewhat surprisingly however, co-expression of this modified peptide precursor, with any of the four AEPs tested, resulted in no detectable cyclic [D14N]SFTI-1. This result contrasts to our *in vitro* assessment of the [D14N]SFTI-1-_GLDN_ substrate where recombinant OaAEP1_b_ predominantly processed the substrate to cyclic [D14N]SFTI-1 (Figure 2D). These results suggest that *in planta*, [D14N]SFTI-1__GLDN_ is cyclizable but is prone to rapid degradation. Interestingly, this does not seem to be the case for kB1, where cyclic peptides containing either asparagine or aspartic acid residues at the kB1 cyclization point accumulate to high levels in *N. benthamiana* leaves (Supplementary Figure S2).

### Plant Produced [T4Y,I7R] Sunflower Trypsin Inhibitor-1 and Synthetically Produced Peptide are Equivalent

**Figure 4:**
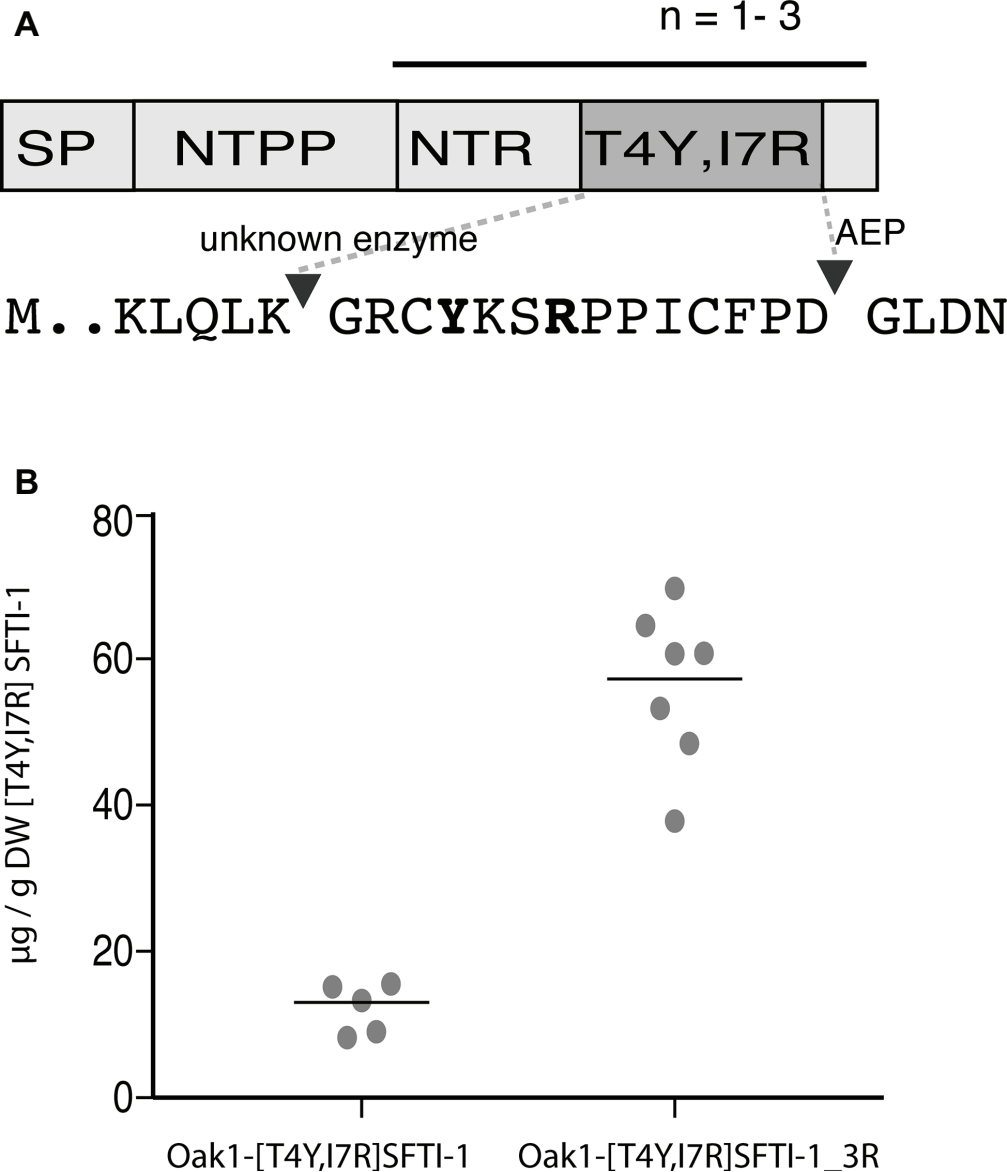
Transient expression analysis of [T4Y,I7R]SFTI-1 production in *N. benthamiana*. **(A)** Two constructs were prepared to produce [T4Y,I7R]SFTI-1 *in planta*. In the first, the kB1 domain normally present in the Oak1 precursor was substituted directly for [T4Y,I7R]SFTI-1. In the second, the amino terminal repeat (NTR), [T4Y,I7R]SFTI-1 and C-terminal tail domain were incorporated as three tandem repeats, adjacent the signal peptide (SP) and amino terminal propeptide (NTPP). **(B)** *In planta* yields (μg/g DW) were calculated using the method of standard addition (Supplementary Figure S3).

The SFTI-1-based plasmin inhibitor [T4Y,I7R]SFTI-1 is the most potent inhibitor of plasmin developed to date (Swedberg et al., 2019). In addition to its high potency (*K*_i_ = 0.041 nM), the inhibitor displays a million-fold selectivity over other serine proteases found in blood and is a promising lead compound for certain antifibrinolytic treatments. As this inhibitor carries only two residue changes to SFTI-1 and retains an aspartic residue for AEP-mediated cyclization, we hypothesized that it is a good candidate for plant-based production. Similar to wild-type SFTI-1 peptide, [T4Y,I7R]SFTI-1 production required the co-expression of the *O. affinis* OaAEP1_b_ ligase (Figure 4A) where yields of 12.3 ± 3.3 μg g^−1^ DW (s.d., *n* = 5) cyclic [T4Y,I7R]SFTI-1 were obtained (Figure 4B, Supplementary Figure S3). As an approach to further improve these yields, we then reengineered the Oak1 precursor to contain three tandem repeats of the [T4Y,I7R]SFTI-1 peptide (Figure 4A). Tandem repeats of kalata type peptides are commonly observed in cyclotide precursor genes (Craik and Malik, 2013) and, at least in part, may be responsible for the observed high yields. By making this change, we improved the *in planta* yield of [T4Y,I7R]SFTI-1 ~5-fold to 56.5 ± 10.8 μg g^−1^ DW (s.d., *n* = 7) (Figure 4B, Supplementary Figure S3).

**Figure 5:**
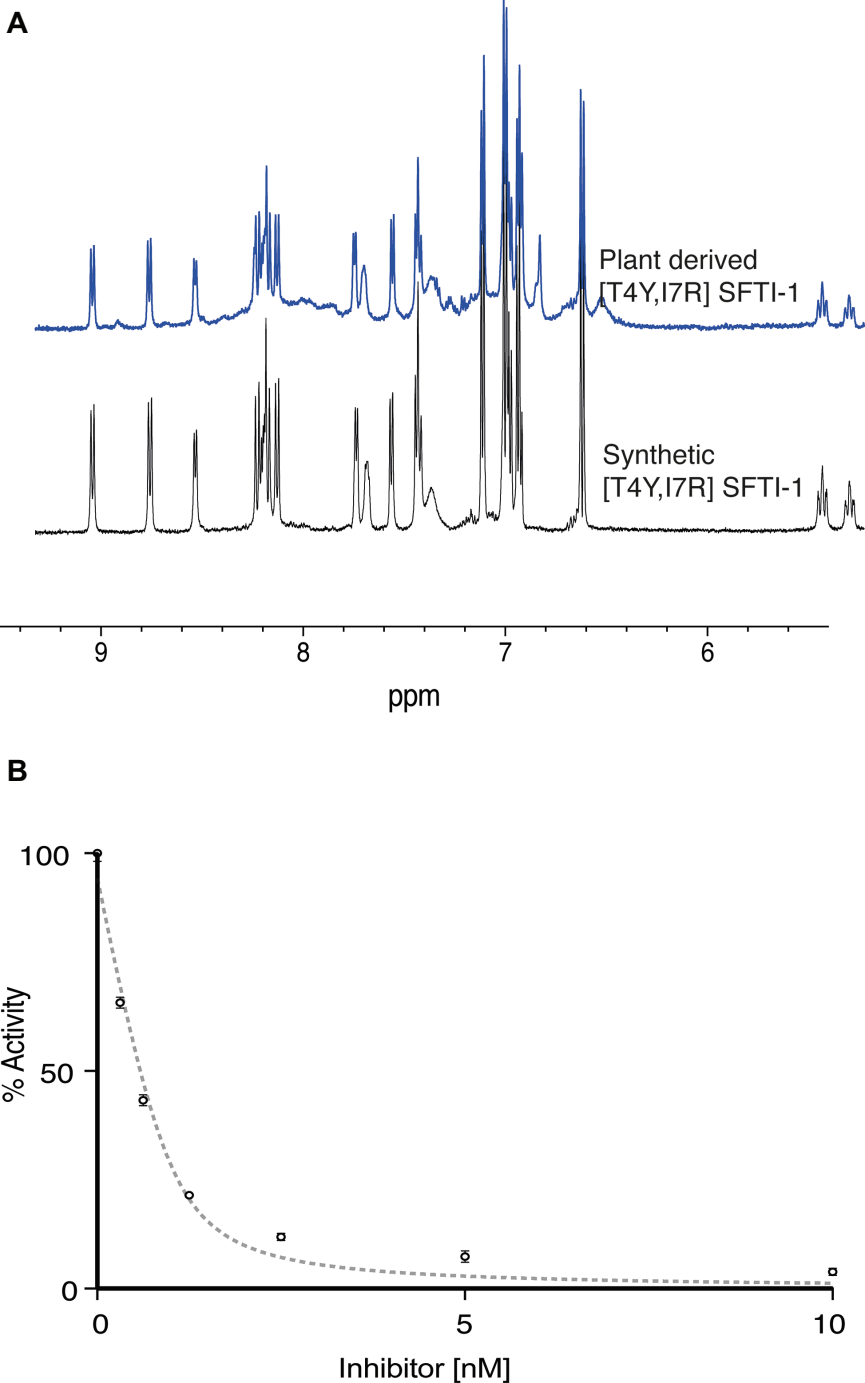
Structural and functional equivalence studies of plant derived vs. synthetic [T4Y,I7R]SFTI-1. **(A)** 1D ^1^H NMR fingerprint region of synthetic and *in planta* produced [T4Y,I7R]SFTI-1. **(B)** Plant-derived [T4Y,I7R]SFTI-1 inhibitor assay (*n* = 3).

To validate the functional and structural equivalence of synthetic peptide vs. the *in planta* produced [T4Y,I7R]SFTI-1, we purified [T4Y,I7R]SFTI-1 peptide from lyophilized leaf tissue. Peptides were extracted, fractionated, and purified using C18 SPE with several rounds of reverse phase HLPC. Structural equivalence to synthetically produced [T4Y,I7R]SFTI-1 was demonstrated *via* LC-MS coelution (Supplementary Figure S4), MS-MS fragmentation patterns (Supplementary Figure S5), and NMR analysis (Figure 5A, Supplementary Figure S6). Functional equivalence of plant-produced [T4Y,I7R]SFTI-1 was demonstrated by determining the inhibitory constant of the purified peptide. The calculated *K*_i_ was 0.025 ± 0.004 nM (Figure 5B), which is comparable to the *K*_i_ 0.041 ± 0.005 nM previously calculated for synthetic peptide (Swedberg et al., 2019).

## Discussion

Peptides as therapeutics are posited to bridge the gap between traditional small molecule drugs and larger biologics, offering the potential of higher specificity, reduced off-target effects, and potentially lower production costs (Craik et al., 2013). However, one drawback is their poor stability, which directly affects efficacy due to shorter *in vivo* half-lives. To counter this limitation, much emphasis has been placed on developing strategies to stabilize peptides, of which backbone cyclization has shown great promise (Poth et al., 2013; Thapa et al., 2014). A recombinant production system that provides for post-translational backbone cyclization of peptides is thus highly desired. Here, we demonstrate a rapid, plant-based approach to produce and cyclize SFTI-1 peptide analogues in an environmentally friendly manner with capacity for scale-up.

In sunflower seed, SFTI-1 is processed from the PawS1 precursor, which additionally encodes for a seed storage albumin that is exclusively found in seed (Figure 1A) (Mylne et al., 2011). For expression in plant leaves, we chose to use the strategy described in (Poon et al., 2017) where the *Oak1* gene was reengineered to include SFTI-1, replacing the kB1 domain (Figure 1B). This ensured that the vacuole targeting elements within Oak1 (Conlan et al., 2011) that work efficiently in *N. benthamiana* leaf cells would be sufficient to direct the engineered SFTI-1 precursor to the vacuole where functional AEP enzymes are believed to reside. An additional benefit of this approach was simplified biosynthesis, as it did not require the concerted action of multiple AEP isoforms, which are required for SFTI-1 maturation from the PawS1 precursor protein (Mylne et al., 2011). The success shown here for a *N. benthamiana* leaf-based SFTI-1 production provides hope that the “plug n play” type approach presented here could be extended to other bioactive peptides derived from seed, such as the trypsin inhibitor class of cyclic peptides derived from *Momordica cochinchinensis* seed (Hernandez et al., 2000).

By using transient gene expression technology, we were able to produce 12.8 ± 3.0 μg g^−1^ DW (s.d., *n* = 3) SFTI-1 upon 6 days of incubation (Figure 1D). Although this is an improvement on the reported natural SFTI-1 levels in mature sunflower seed (0.5 μg g^−1^ seed) (Bernath-Levin et al., 2015), it is lower then that previously reported for the production of the cyclotide kB1, using similar transient gene expression conditions (Poon et al., 2017). By comparing *in vitro* cyclization efficiencies, we demonstrated that this is due to the slower processing of SFTI-1 peptide precursors by OaAEP1_b_ compared to kB1 precursor substrates. One strategy to increase efficiency would be to use the native AEP ligase from sunflower, which may be more efficient for SFTI-1 precursor processing, if expressed heterologously in *N. benthamiana*. However, so far no efficient ligase-type sunflower AEP has been reported (Bernath-Levin et al., 2015; Haywood et al., 2018). Attempts to improve the *in planta* yield of SFTI-1 by co-expressing other known AEP ligases were unsuccessful, with OaAEP1_b_ remaining the superior ligase for SFTI-1. In contrast for kB1, the superior ligase for *in planta* activity is CtAEP1, with OaAEP1_b_ being on par with HeAEP3 (Supplementary Figure S2). These results indicate that substrate preference plays a key role in the *in planta* performance of AEP ligases; however, differences in transcript stability, translational efficiency, enzyme maturation, and stability are also likely.

Plant AEPs are known to have a strict preference for processing at either asparagine or aspartic residues and thus it was surprising that expression of OaAEP1_b_ *in planta* could only produce cyclic SFTI-1 and not [D14N]SFTI-1. We confirmed through *in vitro* cyclization experiments that this was not due to an inefficiency of the enzyme, but more likely due to instability of the precursor or cyclic [D14N]SFTI-1 *in planta*. As only the AEP processing residue was changed, we reasoned that the instability observed is likely governed by the pool of endogenous AEPs present in *N. benthamiana* leaf cells, which may either outcompete the transgene-derived AEP ligase or degrade any correctly cyclized [D14N]SFTI-1. Interestingly, this was not observed with kB1 cyclized with either an asparagine or aspartic residue at the cyclization site (Supplementary Figure S2) and suggests that the observed instability of [D14N]SFTI-1 may be specific to SFTI-1 peptides. This finding is particularly relevant in the case of SFTI-1 peptide analogues that require the D14N residue substitution for potency (Swedberg et al., 2011), in which case, developing strategies to downregulate or knock out endogenous interfering AEPs with emerging gene editing technologies (Puchta, 2017) would be beneficial.

SFTI-1 peptide analogues have been engineered for diverse therapeutic applications ranging from anti-cancer (Swedberg et al., 2009, 2011), anti-obesity (Durek et al., 2018), pro and anti-angiogenesis (Chan et al., 2011; Qiu et al., 2017) as well as for treatment of a range of skin conditions (Chen et al., 2016; Zhu et al., 2017). Here, we have shown that like SFTI-1, the engineered plasmin inhibitor [T4Y,I7R]SFTI-1 (Swedberg et al., 2019) is amenable to plant-based production with the resultant purified peptide displaying both structural and functional equivalence to synthetically produced material. Through expression of a multi peptide domain gene construct the yield of the 14 amino acid cyclic [T4Y,I7R]SFTI-1 reached ~60 μg/g DW, roughly equivalent to that obtained previously for kB1 (29 aa) on a molar basis. Importantly, this approach provides for economies of scale, with lower inputs and infrastructure costs than synthetic peptide synthesis. Although currently still an emerging industry, commercial facilities for plant-based recombinant production have begun to be established, primarily for vaccine production. One such facility operated by iBio Biotherapeutics has a reported capacity to process ~3,500 kg of plant material per week (Holtz et al., 2015). Although, the economics of scaling up a plant-based production approach for peptide therapeutic production must be considered on a case-by-case basis, backbone cyclized peptide scaffolds such as SFTI-1 represent a particularly suitable case, given their natural occurrence in plants.

## Author Contributions

MJ, EG, TD, KH, DC, and MA conceived the experiments. MJ, SP, and HQ made gene constructs and performed transient assays. KY and AP performed MS analysis. KY and JS performed functional analysis. TD performed NMR analysis. KH and KY produced and assayed recombinant AEP. All authors contributed to the writing of the manuscript.

### Conflict of Interest Statement

The authors declare that the research was conducted in the absence of any commercial or financial relationships that could be construed as a potential conflict of interest.
