# Supplementary material for: Rapid and Scalable Plant-Based Production of a Potent Plasmin Inhibitor Peptide
Source: Front Plant Sci. 2019 May 15;10:602. doi: 10.3389/fpls.2019.00602 (PMC6530601; doi:10.3389/fpls.2019.00602)
Supplement: Supplementary file 1 [file Data_Sheet_1.PDF]

Supplementary Information

Jackson et al. **Rapid and scalable plant based production of a potent plasmin inhibitor peptide**

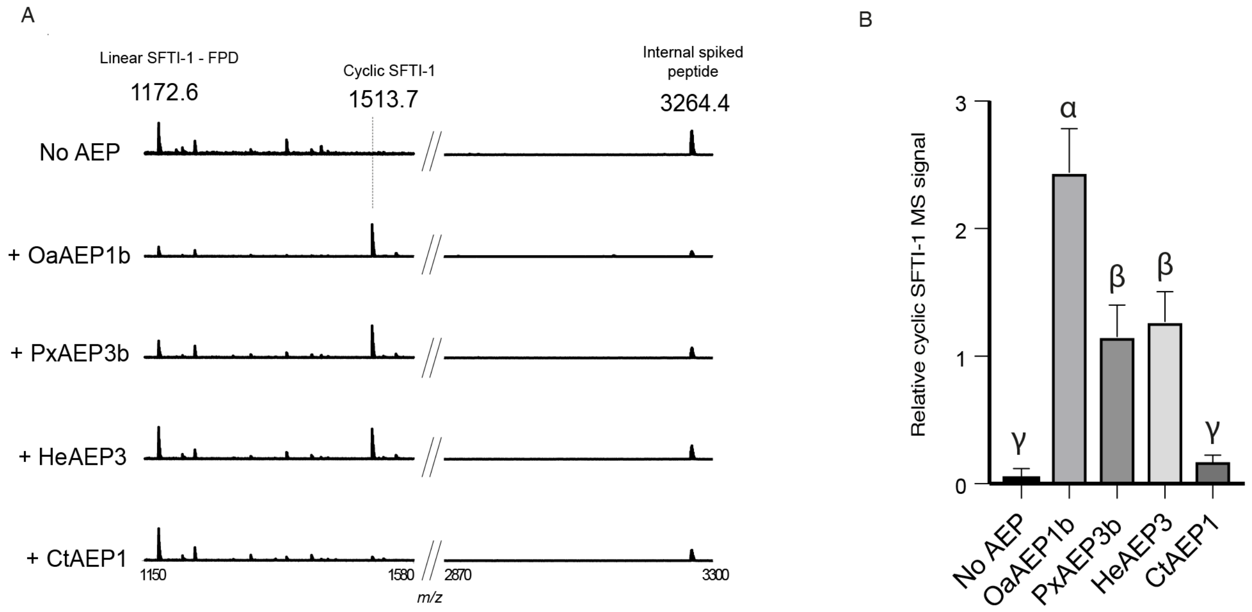

Figure S1. Comparison of AEP ligases for the *in planta* SFTI-1 peptide cyclisation. (A) MALDI-TOF MS analysis of representative (n=6) peptide extracts from *N. benthamiana* leaves which were co-infiltrated with pEAQ-Oak1-SFTI1_GLDN and AEP ligase genes. Without AEP transgene expression, no cyclic or full length linear SFTI-1 related peptides were detectable. Smaller masses, however were observed, with low signal strengths, and likely represent truncated SFTI-1 peptides (e.g *m/z* 1172.6 consistent with linear oxidised GRCTKSIPPIC. By co-expressing AEP ligase genes from *O. affinis* (OaAEP1_b), *H. enneaspermus* (HeAEP3) and Petunia 'Mitchell' (PxAEP3b) cyclic SFTI-1 was readily detected which contrasted to expression of CtAEP1 which failed to produce any cyclic SFTI-1. **(B)** Relative MS signal intensities for cyclic SFTI-1 among co-expressed ligase capable AEPs (n=6). For relative quantifications, a spiked peptide control (*m/z* 3264.4) was added on a per gram DW basis. Treatments carrying unique Greek lettering are significantly different ($P<0.05$) as determined by Tukey's ANOVA. Error bars are s.e.m.

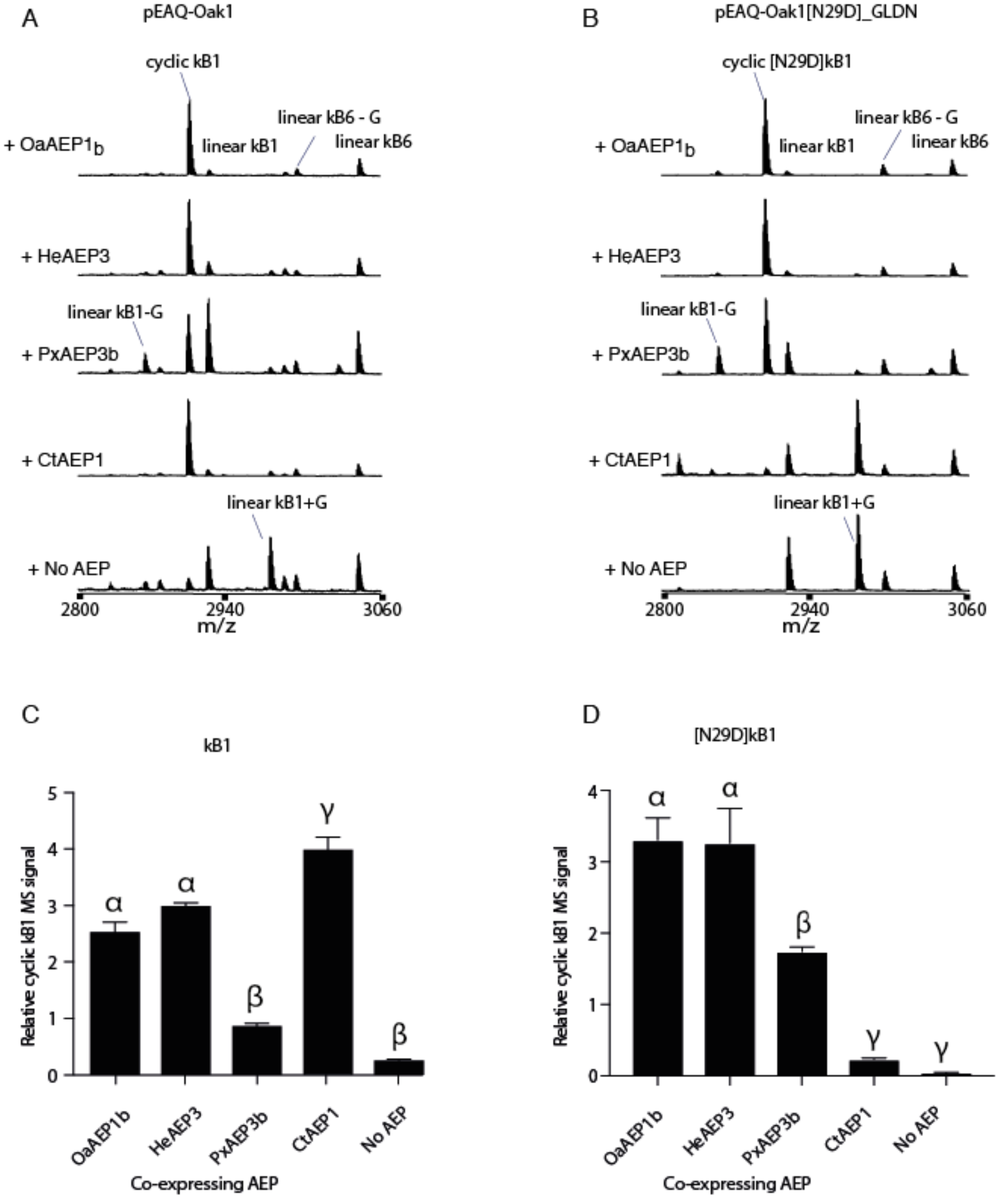

Figure S2. Comparison of AEP ligases for the *in planta* cyclisation of kB1. MALDI-TOF MS analysis of representative (n=3) peptide extracts from *N. benthamiana* leaves which were co-infiltrated with **(A)** pEAQ-Oak1, pEAQ-Oak6trun with or without pEAQ expressing AEP ligase genes or **(B)** pEAQ-Oak1[N29D]_GLDN, pEAQ-Oak6trun with or without pEAQ expressing AEP ligase genes. For all infiltrations, the expression of pEAQ-Oak6trun served as an internal control where MS signal intensities for linear kB6 were used to normalise kB1 signals for relative quantification. Relative cyclic kB1 **(C)** and kB1[N29D] **(D)** levels respectively calculated upon co-expression of ligase-capable AEPs (n=3). Treatments carrying unique Greek lettering are significantly different (P<0.05) as determined by Tukey's ANOVA. Error bars are s.e.m.

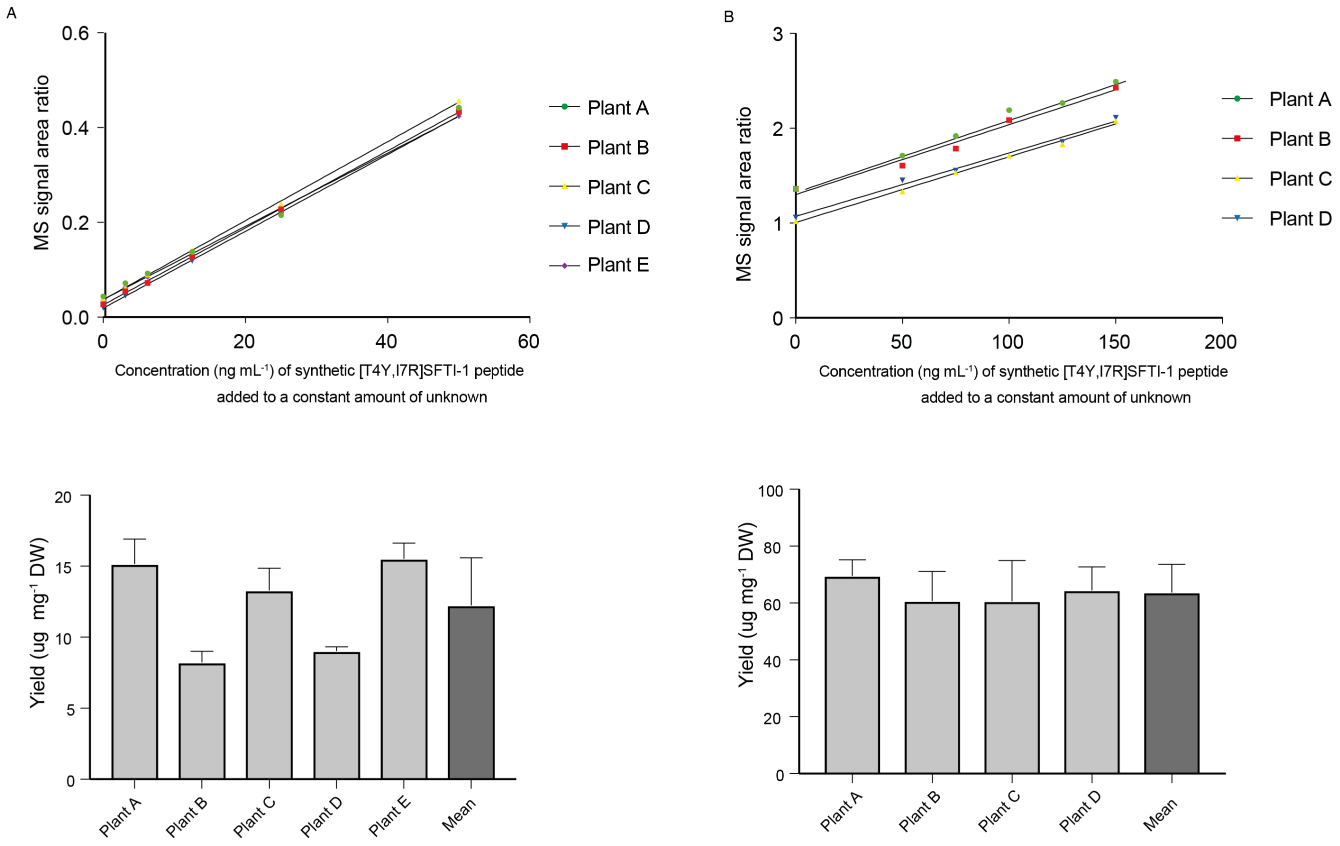

Figure S3. Method of standard addition approach for quantifying [T4Y,I7R] SFTI-1 levels in *N. benthamiana* leaf extracts. Quantification of [T4Y,I7R] SFTI-1 yields after co-expression of (a) pEAQ-Oak1_[T4Y,I7R] SFTI-1 with pEAQ_OaAEP1$_b$ (n=5) and (b) pEAQ-Oak1_[T4Y,I7R] SFTI-1-3R with pEAQ_OaAEP1$_b$ (n=4).

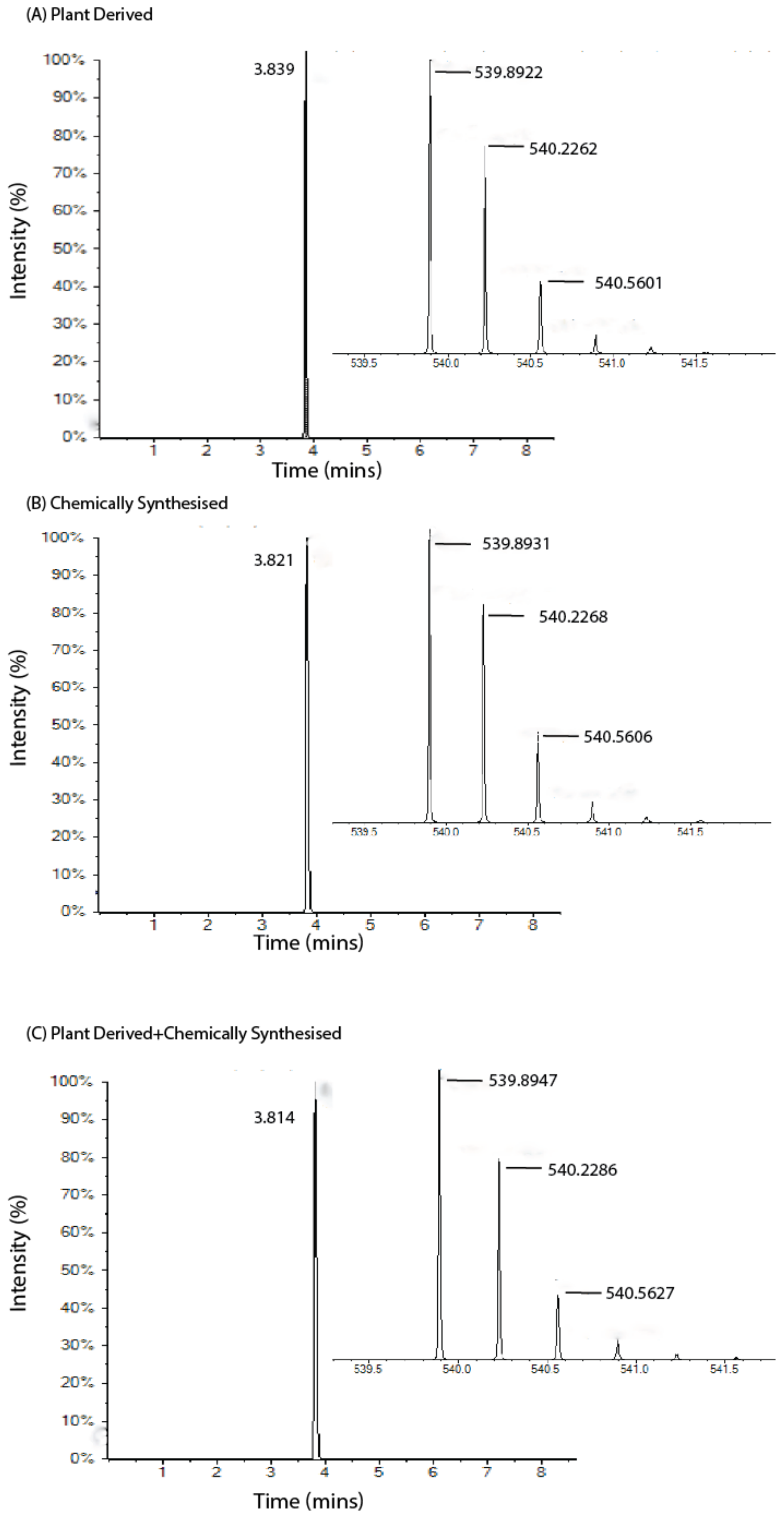

Figure S4. LC-MS co-elution studies of plant-derived and synthetic [T4Y,I7R] SFTI-1. (A) XIC and ESI-TOF MS spectrum of plant-derived [T4Y,I7R] SFTI-1. **(B)** synthetic [T4Y,I7R] SFTI-1. **c.** a 1:1 mixture of plant-derived and synthetic [T4Y,I7R] SFTI-1.

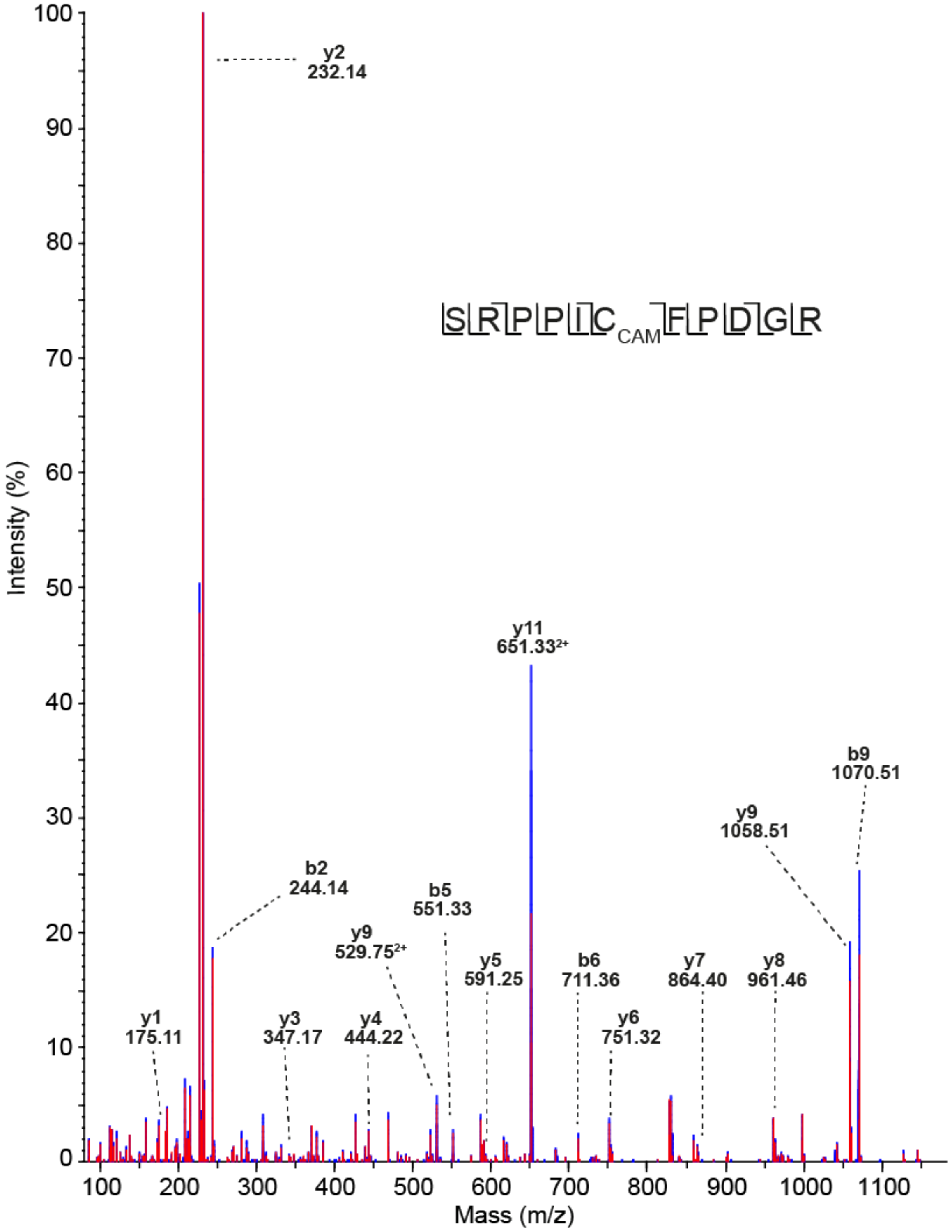

Figure S5. MS/MS analysis of *m/z* 651.3^{2+}, a tryptic fragment of reduced and alkylated [T4Y,I7R] SFTI-1.

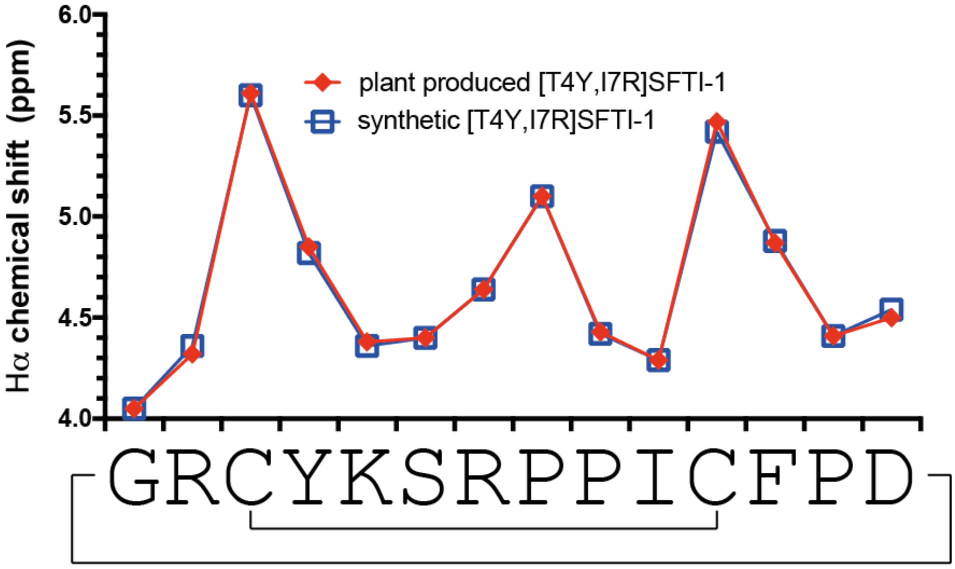

Figure S6. Hα – chemical shift comparison of [T4Y,I7R] SFTI-1 produced by solid phase peptide chemistry (synthetic) with the same peptide purified from *N. benthamiana* leaf.

Oak1-SFTI-1_GLDN

```
ATGGCTAAGTTCACCGTCTGTCTCCTCCTGTGCTTGCTTCTTGCAGCATTTGTTGGGGCGTTTGGATCTGAGCTTTCTGACTCCCACAAGACCACCTTGG
 M  A  K  F  T  V  C  L  L  L  C  L  L  L  A  A  F  V  G  A  F  G  S  E  L  S  D  S  H  K  T  T  L>

TCAATGAAATCGCTGAGAAGATGCTACAAAGAAAGATATTGGATGGAGTGGAAGCTACTTTGGTCACTGATGTCGCCGAGAAGATGTTCCTAAGAAAGAT
V  N  E  I  A  E  K  M  L  Q  R  K  I  L  D  G  V  E  A  T  L  V  T  D  V  A  E  K  M  F  L  R  K  M>
                                                                              |
GAAGGCTGAAGCGAAAACTTCTGAAACCGCCGATCAGGTGTTCCTGAAACAGTTGCAGCTCAAAGGAAGATGTACCAAGTCTATCCCTCCTATCTGTTTC
  K  A  E  A  K  T  S  E  T  A  D  Q  V  F  L  K  Q  L  Q  L  K  G  R  C  T  K  S  I  P  P  I  C  F>

CCTGATGGCCTTGATAATTAATGA
 P  D  G  L  D  N  *  *>
```

Oak1-SFTI-1_NGLDN

```
ATGGCTAAGTTCACCGTCTGTCTCCTCCTGTGCTTGCTTCTTGCAGCATTTGTTGGGGCGTTTGGATCTGAGCTTTCTGACTCCCACAAGACCACCTTGG
 M  A  K  F  T  V  C  L  L  L  C  L  L  L  A  A  F  V  G  A  F  G  S  E  L  S  D  S  H  K  T  T  L>

TCAATGAAATCGCTGAGAAGATGCTACAAAGAAAGATATTGGATGGAGTGGAAGCTACTTTGGTCACTGATGTCGCCGAGAAGATGTTCCTAAGAAAGAT
V  N  E  I  A  E  K  M  L  Q  R  K  I  L  D  G  V  E  A  T  L  V  T  D  V  A  E  K  M  F  L  R  K  M>
                                                                              |
GAAGGCTGAAGCGAAAACTTCTGAAACCGCCGATCAGGTGTTCCTGAAACAGTTGCAGCTCAAAGGAAGATGTACCAAGTCTATCCCTCCTATCTGTTTC
  K  A  E  A  K  T  S  E  T  A  D  Q  V  F  L  K  Q  L  Q  L  K  G  R  C  T  K  S  I  P  P  I  C  F>

CCTAATGGCCTTGATAATTAATGA
 P  N  G  L  D  N  *  *>
```

Oak1-GLDN

```
ATGGCTAAGTTCACCGTCTGTCTCCTCCTGTGCTTGCTTCTTGCAGCATTTGTTGGGGCGTTTGGATCTGAGCTTTCTGACTCCCACAAGACCACCTTGG
 M  A  K  F  T  V  C  L  L  L  C  L  L  L  A  A  F  V  G  A  F  G  S  E  L  S  D  S  H  K  T  T  L>

TCAATGAAATCGCTGAGAAGATGCTACAAAGAAAGATATTGGATGGAGTGGAAGCTACTTTGGTCACTGATGTCGCCGAGAAGATGTTCCTAAGAAAGAT
V  N  E  I  A  E  K  M  L  Q  R  K  I  L  D  G  V  E  A  T  L  V  T  D  V  A  E  K  M  F  L  R  K  M>

GAAGGCTGAAGCGAAAACTTCTGAAACCGCCGATCAGGTGTTCCTGAAACAGTTGCAGCTCAAAGGACTTCCAGTATGCGGTGAGACTTGTGTTGGGGGA
  K  A  E  A  K  T  S  E  T  A  D  Q  V  F  L  K  Q  L  Q  L  K  G  L  P  V  C  G  E  T  C  V  G  G>

ACTTGCAACACTCCAGGCTGCACTTGCTCCTGGCCTGTTTGCACACGCAATGGCCTTGATAATTAA
 T  C  N  T  P  G  C  T  C  S  W  P  V  C  T  R  N  G  L  D  N  *>
```

Oak1[N29D]-GLDN

```
ATGGCTAAGTTCACCGTCTGTCTCCTCCTGTGCTTGCTTCTTGCAGCATTTGTTGGGGCGTTTGGATCTGAGCTTTCTGACTCCCACAAGACCACCTTGG
 M  A  K  F  T  V  C  L  L  L  C  L  L  L  A  A  F  V  G  A  F  G  S  E  L  S  D  S  H  K  T  T  L>

TCAATGAAATCGCTGAGAAGATGCTACAAAGAAAGATATTGGATGGAGTGGAAGCTACTTTGGTCACTGATGTCGCCGAGAAGATGTTCCTAAGAAAGAT
V  N  E  I  A  E  K  M  L  Q  R  K  I  L  D  G  V  E  A  T  L  V  T  D  V  A  E  K  M  F  L  R  K  M>

GAAGGCTGAAGCGAAAACTTCTGAAACCGCCGATCAGGTGTTCCTGAAACAGTTGCAGCTCAAAGGACTTCCAGTATGCGGTGAGACTTGTGTTGGGGGA
  K  A  E  A  K  T  S  E  T  A  D  Q  V  F  L  K  Q  L  Q  L  K  G  L  P  V  C  G  E  T  C  V  G  G>

ACTTGCAACACTCCAGGCTGCACTTGCTCCTGGCCTGTTTGCACACGCGATGGCCTTGATAATTAA
 T  C  N  T  P  G  C  T  C  S  W  P  V  C  T  R  D  G  L  D  N  *>
```

Oak1-[T4Y,I7R]SFTI-1

```
ATGGCTAAGTTCACCGTCTGTCTCCTCCTGTGCATGCTTCTTGCAGCATCTGCAGGGGCGTTTGGATCTGAGCGTTGTGACTCCCACAAGACCACCTTGG
 M  A  K  F  T  V  C  L  L  L  C  M  L  L  A  A  S  A  G  A  F  G  S  E  R  C  D  S  H  K  T  T  L>

TCAATGAAATCGCGGAGAAGATGCTACAAAGAAAGATATTGGATGGAGTGGAAGCTACTTTGGTCACTGATGTCGCCGAGAAGATGTTCCTAAGAAAGAT
V  N  E  I  A  E  K  M  L  Q  R  K  I  L  D  G  V  E  A  T  L  V  T  D  V  A  E  K  M  F  L  R  K  M>

GAAGGCTGAAGCGAAAACTTCTGAAACCGCCGATCAGGTGTTCATGAAACAGTTGCAGCTCAAAGGAAGATGTTACAAGTCTAGACCTCCTATCTGTTTC
  K  A  E  A  K  T  S  E  T  A  D  Q  V  F  M  K  Q  L  Q  L  K  G  R  C  Y  K  S  R  P  P  I  C  F>

CCTGATGGCCTTGATAATTAA
 P  D  G  L  D  N  *>
```

Oak1-[T4Y,I7R]SFTI-1_3R

```
ATGGCTAAGTTCACCGTCTGTCTCCTCCTGTGCATGCTTCTTGCAGCATCTGCAGGGGCGTTTGGATCTGAGCGTTGTGACTCCCACAAGACCACCTTGG
 M  A  K  F  T  V  C  L  L  L  C  M  L  L  A  A  S  A  G  A  F  G  S  E  R  C  D  S  H  K  T  T  L>

TCAATGAAATCGCGGAGAAGATGCTACAAAGAAAGATATTGGATGGAGTGGAAGCTACTTTGGTCACTGATGTCGCCGAGAAGATGTTCCTAAGAAAGAT
V  N  E  I  A  E  K  M  L  Q  R  K  I  L  D  G  V  E  A  T  L  V  T  D  V  A  E  K  M  F  L  R  K  M>

GAAGGCTGAAGCGAAAACTTCTGAAACCGCCGATCAGGTGTTCATGAAACAGTTGCAGCTCAAAGGAAGATGTTACAAGTCTAGACCTCCTATCTGTTTC
  K  A  E  A  K  T  S  E  T  A  D  Q  V  F  M  K  Q  L  Q  L  K  G  R  C  Y  K  S  R  P  P  I  C  F>

CCTGATGGCCTTGATAATATGAAGGCTGAAGCAAAAACTTCAGAGACTGCTGATCAAGTTTTTCTTAAGCAGTTGCAACTTAAGGGTAGATGTTATAAGT
 P  D  G  L  D  N  M  K  A  E  A  K  T  S  E  T  A  D  Q  V  F  L  K  Q  L  Q  L  K  G  R  C  Y  K>

CAAGGCCTCCAATTTGTTTCCCAGATGGTCTTGATAATATGAAAGCAGAGGCTAAGACATCTGAAACAGCAGACCAGGTGTTCTTGAAACAACTTCAGTT
S  R  P  P  I  C  F  P  D  G  L  D  N  M  K  A  E  A  K  T  S  E  T  A  D  Q  V  F  L  K  Q  L  Q  L>

GAAAGGAAGGTGCTACAAATCTAGACCACCTATATGCTTTCCTGACGGATTGGACAACTAA
  K  G  R  C  Y  K  S  R  P  P  I  C  F  P  D  G  L  D  N  *>
```

Figure S7. Nucleotide and deduced amino acid sequences of peptide precursor genes used in this study.

Table S1. MRM transitions for analytes in quantitation studies

Analyte	Q1 (*m/z*)	Q3 (*m/z*)	CE (V)	DP (V)
[T4Y,I7R]SFTI-1-pseudoMRM	540.4	540.5	5.0	87.0
[T4Y,I7R]SFTI-1 Qualifier	540.4	736.5	30.9	92.0
Codeine-ISTD	300.1	300.2	5.0	130.0
